# All-in-One Mosquito Containers: From the Laboratory to the Release Sites

**DOI:** 10.3390/insects13020178

**Published:** 2022-02-09

**Authors:** Carlos Tur, Ignacio Plá, Rafael Argilés-Herrero, Gustavo Salvador-Herranz, David Almenar

**Affiliations:** 1 Empresa de Transformación Agraria S.A., S.M.E., M.P. (TRAGSA). Avenida de la Industria 26, 46980 Paterna, Spain; ipla@tragsa.es (I.P.); dalmenar@tragsa.es (D.A.); 2 Escuela de Doctorado, Universidad Católica de Valencia San Vicente Mártir, 46001 Valencia, Spain; 3 Insect Pest Control Section, Joint FAO/IAEA Division of Nuclear Techniques in Food and Agriculture, Wagramerstrasse 5, P.O. Box 100, A-1400 Vienna, Austria; rargiles@hotmail.com; 4 Technical School of Design, Architecture and Engineering, University CEU Cardenal Herrera, C/San Bartolomé 55, 46115 Alfara del Patriarca, Spain; gsalva@uchceu.es

**Keywords:** culicidae, Sterile Insect Technique (SIT), genetic control of vectors, release, container, automated

## Abstract

**Simple Summary:**

The Asian tiger mosquito, *Aedes albopicus* (Skuse), is a vector of dangerous diseases, such as dengue, Zika and Chikungunya, and has spread extensively in Europe since its initial detection in the 1970s. The Sterile Insect Technique (SIT) has proven effective in disease-vector pilot projects carried out worldwide by producing and releasing high quality, sterile male mosquitoes. To implement the Sterile Insect Technique in large-scale programs, improved cost-effectiveness would be required. In the following article we will assess the effect that containing sterile male mosquitoes in the proposed all-in-one containers from pupa to adult stages and until release has on their quality. We will also assess the potential damage to samples during handling and how production costs could be reduced. An automatic variable-dosage release system, currently under development, to be installed in publicly-owned vehicles, may release sterile mosquitoes in the appropriate dose, optimizing available resources and considerably reducing the release cost.

**Abstract:**

Integrated vector control programs that use a Sterile Insect Technique approach require the production and release of large numbers of high quality, sterile male insects. In pilot projects conducted worldwide, sterile males are usually kept in containers at low densities until their manual release on the ground. Although the quality of the released insects is high, these containers are only suitable for small-scale projects, given the fact that the manual labor required for release is significant and therefore untenable in large-scale projects. This study will compare and contrast the quality of the males reared in the proposed “all-in-one” containers which considerably reduce both the handling of the insects and the manual labor required for release. As a result, project costs are lower. The design of these “all-in-one” containers incorporates two important features: ventilation and the density of the vertical resting surface. Having evaluated both features, it can be concluded that ventilation does not directly affect the quality of the insects, at least in the range of dimensions tested. However, the quality of the male insects is reduced in relation to an increase in the number of mosquitoes, with 500 being the optimum quantity of mosquitoes per “all-in-one” container.

## 1. Introduction

The increase in incidence of vector-borne diseases in recent decades is an international concern, and the ability to combat them a global challenge for humanity [1]. Governments and both private and state-owned national and international institutions are promoting and funding research projects to find solutions in the fight against vectors. Vector control of mosquitoes, flies and bugs, is considered a key public health measure [1]. In recent years, there has been a significant increase in the number of imported cases of Dengue, Zika and Chikungunya in Europe. This correlates with a general rise in global incidence and an increase in the number of travelers to and from tropical areas. *Aedes albopictus* (Skuse) is a well-known vector of these diseases, present in Europe since 1970s, having been first reported in 1979 in Albania [2]. In the last three decades, this species of mosquito has mostly spread [3] to new areas via passive transportation—on tires, etc.—and has been now been detected worldwide. Current incidence spans many areas of Europe, namely, Spain, France, Italy, Greece, etc. [4].

Traditional control strategies based on the use of pesticides are insufficient due to the fact that *Ae. albopictus* is a highly adaptive species with a great capacity for proliferation. Many ideal breeding sites exist, which are often private, hidden and inaccessible, in which females can lay hundreds of eggs. Similarly, mosquitoes have developed resistance to certain types of insecticides which are consequently rendered ineffective [5]. As such, an Integrated Vector Management (IVM) strategy as a “rational decision-making process for the optimal use of resources for vector control” in which “the approach seeks to improve the efficacy, cost-effectiveness, ecological soundness and sustainability of disease-vector control” [6] is considered to be the most effective method of *Ae. albopictus* control.

The Sterile Insect Technique (SIT) is a pest control method that consists of mass-rearing male insects of the same species which are sterilized by irradiation and released into the field. The sterile males mate with wild females, which then lay non-viable eggs [7]. It is an autocidal control technique which respects the environment, human health, and food; does not promote resistance; and has proven effective in controlling pests in projects around the world for decades [8,9,10].

In recent years, research projects focused on the application of SIT against mosquitoes have been promoted by both private and public institutions [11,12,13,14,15]. Progress has been noted in relation to the development of technology, equipment and procedures to increase the viability of SIT, from both technical and economic perspectives and in medium and large-scale projects. Recent advances in the development of automatic mosquito sex sorting systems [16,17] are helping to effectuate the application of SIT to real settings.

With regard to the application of SIT to control *Aedes* spp. mosquitoes, several successful small-scale projects have been carried out in recent years. This demonstrates the technical feasibility of SIT control of this specific pest [11,17,18]. From 2018 to the present day, more than 15 million *Ae. albopictus* sterile mosquitoes have been successfully released in an area of 80 ha. in the Valencian Region of Spain. The results of this pilot project, led and financed by the Department of Agriculture of Valencia and carried out by Grupo TRAGSA, demonstrate the technical feasibility of SIT to combat the vector (Tur et al. *in prep).* Notwithstanding, there remains a two-fold need to further develop equipment, technology and procedures. Firstly, in order to ensure this technique becomes an economically viable method of mosquito control, and secondly, to guarantee the current quality of insects produced and released. Low temperature collection and minimized handling levels of mosquitoes are currently under discussion at the international level. They are, per se, fragile insects and easily damaged during handling (broken legs, wings or other parts of the body). Furthermore, the length of exposure to low temperatures could affect their quality [19,20]. Consequently, a rearing, transport and release method that avoids chilling and reduces handling may result in higher quality male mosquitoes.

Empirical evidence gained through the design and development of handling cages for *C. capitata* sterile males shows that ventilation and the vertical Density Resting Surface (DRS) are the two features that most influence the quality of the insects. The DRS is a parameter that measures the number of insects per area available for resting, obtained by dividing the number of mosquitoes per vertical unit area (mosquitoes/cm^2^). In this research, these two features were studied during the process of testing using the “all-in-one” (AIO) containers developed by Grupo TRAGSA and MAPA TECHNOLOGY S.L. within the framework of the Coordinated Research Project D44002 “Mosquito Handling, Transport, Release and Male Trapping Methods” promoted by the Food and Agriculture Organization of the United Nations (FAO) and the International Atomic Energy Agency (IAEA). After the pupae were sorted according to sex, male mosquitoes were kept within these AIO containers until release, eliminating a possible reduction in quality due to cooling or handling. The irradiation of the mosquitoes in the pupa stage or adult stage is also possible. Furthermore, the use of this device is considerably less labor-intensive, thereby reducing the current production costs. The AIO containers can be easily automated throughout the process from the initial loading of the pupae to the moment of release. The adult mosquitoes are released into the action area through the installation of ground release machines attached to private or public vehicles such as taxis and buses. The mosquitoes are pushed out of the containers by electronically-controlled pistons. The AIO container has been specifically designed to reduce costs involved in the production and release of mosquitoes. This study aims to evaluate the optimal dimensions and design of the AIO container which preserve the quality of mosquitoes.

## 2. Materials and Methods

### 2.1. Rearing and Biological Material

The mosquito colony was originated from eggs collected from different locations across the Valencian Region of Spain in 2014. The strain was maintained using standard laboratory procedures [21] until 2017, when a SIT pilot requiring the mass-rearing of mosquitoes began [22].

The colony was maintained in several rooms at 25 ± 1.5 °C and 70 ± 10% relative humidity, where the adults were reared in 40 × 40 × 40 cm cages made of methacrylate and polyethylene mesh. Each cage was filled with 10.000 immature mosquitoes (L4 and pupal stages) resulting from sex-sorting, at an estimated female to male ratio of approximately 3:1. Adults were fed with a sucrose-based winter food for bees (Apicomin Jarabe Denso, Kessler Iberica, Montserrat, Spain) and blood meals were given daily to females from the sixth day, using collagen casings (FIBRAN S.A., Girona, Spain) with defibrinated fresh pig blood from an authorized slaughterhouse in accordance with current EU legislation. Several removable pieces of filter paper partially submerged in water were introduced into a container and placed inside the cages for egg collection. These pieces were allowed to dry in partially-closed trays. Eggs older than one week were hatched in a nutrient broth solution [21]. The hatched larvae were subsequently counted [23] in batches of 10,000 and transferred to 40 × 60 cm plastic trays containing 5 L of water where were fed with IAEA larval diet [24] until pupation. Female pupae, male pupae and larvae were separated using a Fay–Morlan apparatus [25].

### 2.2. Evaluation of Container Suitability

**Figure 1:**
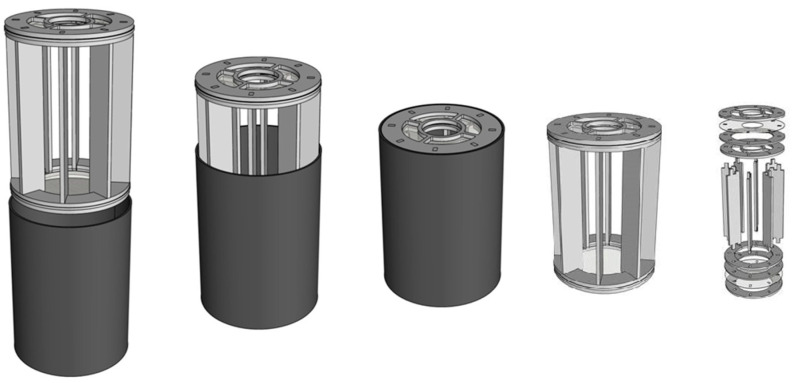
Two-piece tube container featuring an external piece: pipe connection socket; and an internal piece: two methacrylate rings joined together by eight methacrylate strips.

The AIO container is a two-piece tubular design aimed to facilitate automated release. The external piece is a PVC pipe connection socket (8.8 cm inner diameter, 12.5 cm high) featuring a sanded inner surface. The internal piece fits snugly inside the external one and consist of two methacrylate rings joined together by eight methacrylate 11.5 × 2 cm strips that forms a cylindrical-shaped piece to maximize the DRS (Figure 1). The strips are covered with anti-slip stickers, to increase the surface roughness of methacrylate. A 0.7 × 0.3 mm High-density polyethylene Mesh (Beniagro, Benigànim, Spain), is used to seal the hole made by the rings with a remaining 25 mm diameter hole in the upper ring. This hole is covered with a sugar feeder, made of a 50 mL centrifuge tube cut at the 30 mL mark and a cylindrical piece of polyvinyl alcohol sponge (CTS, Altavilla Vicentina, Italy). The tube is filled with a 10% sucrose solution and can be inverted without spillage.

To evaluate the suitability of the container, several consecutive trials were conducted under differing conditions and designs.

In all trials conducted, the density of mosquitoes in the container was calculated according to the density of vertical resting surface (DRS): the number of pupae loaded multiplied by a correction factor of 0.95 (approximated emergence rate from pupae to adult), divided by the area of usable vertical resting surfaces, in mosquitoes/cm^2^ [26].

### 2.3. Experiment 1: To Evaluate the Effect of Ventilation on the AIO Container’s External Piece

In order to test the necessity of lateral ventilation, the performances of two differing containers were compared. The low-ventilation container tube consisted of a piece of PVC pipe (11 cm inner diameter by 12 cm high) with sanded inner surfaces to increase roughness and allow the mosquitoes to rest. In this design, the only ventilation entrance was the upper aperture, opened and closed with a removable piece of PVC mesh (1 mm mesh size). The other ventilated container used consisted of a piece of a screen irrigation filter of the same dimensions, made of stainless-steel mesh (125 micron mesh, sealed with a removable piece of PVC mesh (1 mm mesh size).

Both containers were loaded with 450 male (DRS = 1.1 mosquitoes/cm^2^) pupae through the upper opening 16 h after sex-sorting. The tubes were partially submerged in a vertical position within a tray containing 2 cm-deep water to enable the pupae to swim. A piece of sponge soaked in 10% sucrose solution was then placed externally on the top mesh. Three days after pupa loading, the tubes were removed from the water and left to drain in a grid tray. Five days after the pupae were inserted, a release of adult mosquitoes was simulated by opening the tubes in a polypropylene container (size 17 × 18 × 28 cm) with two 20 × 10 cm windows on the sides covered with PVC mesh (1 mm mesh size). Those males remaining in the tube container were forced out by blowing through the base. Three identical containers were used per treatment.

Mortality during containment was calculated as the percentage of dead males counted in the tube containers after disassembly in relation to the number of pupae initially loaded. The adult males were kept without access to water or sugar in the polypropylene container from which they were released. The proportion of live mosquitoes after 24 h from the total number released was used as an estimate of survival under stress.

### 2.4. Experiment 2: To Evaluate the Effect of Density

In order to evaluate the effect of density, different AIO containers were loaded with 500, 750, 1000 or 1250 pupae (DRSs of 0.69, 1.04, 1.32 and 1.73 mosquitoes/cm^2^ respectively), three containers being used per density. The containers were placed vertically in a tray containing 2 cm-deep water. Three days after the pupae were loaded, the containers were removed from the water tray and left to drain in a grid tray.

Mortality during containment was estimated as the percentage of dead males counted inside the tube containers and control containers on disassembly, in relation to the original number of pupae initially loaded. One hundred of the released males were transferred to a new polypropylene container (identical to the above-mentioned). After 24 h without access to water or sugar, the proportion of dead mosquitoes in relation to the total number transferred was used as an estimate of survival under stress.

A control release container was used for comparison purposes. This release container has been used in routine male releases as part of the SIT pilot project. It is identical in characteristics to the polypropylene container described above (DRS = 0.46 mosquitoes/cm^2^), comprising a 125 mL container with 10% sucrose solution and cellulose sponge. Pupae were inserted inside the container using an 11 cm diameter plastic cup, which was then removed two days later. The same parameters were used throughout for both the control and tested device, and mortality during containment included the dead adults found in the cup. Three replicates were used per control.

### 2.5. Experiment 3: Validation of the Chosen Design

The prime objective of this experiment was to test whether the quality of the mosquitos kept in the proposed design and density (Experiment 2) was equivalent to the quality obtained in the manual release containers used in the present SIT pilot project. Two features were modified: (1) The anti-slip stickers on the vertical methacrylate strips were replaced by sanding; and (2) based on the results of Experiment 2, the containers were left submerged in water for two days instead of three to try to reduce adult mortality.

Three AIO containers were loaded with 500 pupae, as per Experiment 2. Three control containers, similar to the ones described above, were also loaded with 750 pupae from the same batch. Pupae number was estimated volumetrically [21,27] to simulate real mass-rearing conditions. Mortality during containment was assessed as per Experiment 2. In addition, a longevity test was performed for quality assurance. Five days after pupa loading, approximately fifty adult males per treatment were transferred to a 17 × 18 × 28 cm polypropylene container with two 20 × 10 cm windows on the sides covered with PVC mesh (1 mm mesh size) comprising a 125 mL container, with 10% sucrose solution and cellulose sponge. The sugar feeder was replaced with a new one on Day 15. Dead males were counted daily for 24 days. Surviving males at 24 days were classified as “censored” and those having accidentally escaped or suffered damage.

### 2.6. Statistical Analysis

All data were analyzed with R 4.0.3 [28].

Mortality during containment and survival under stress were treated as binary variables and analyzed using mixed model logistic regression. Treatment was employed as a fixed effects predictor variable and the replicate was introduced as a random variable. The R libraries “lme4“, “effects” and “emmeans” were used to fit the models, for effect estimation and to compare marginal means. The longevity of the mosquitoes was analyzed by means of a mixed models Cox proportional hazards model, using the R libraries “survival” and “coxme.” Treatment was employed as a fixed effects predictor variable and the replicate was introduced as a random variable.

## 3. Results

### 3.1. Experiment 1: To Evaluate the Effect of Ventilation on the AIO Container’s External Piece

The average mortality during confinement was slightly lower in the ventilated container than the sealed one (6.95%; SD = 3.9 and 7.08%; SD = 2.29 respectively), although this was not significant (difference = −0.09 ± 0.36, z = −0.279, *p* = 0.781). In relation to the released adults, average survival under stress was higher for those adults released from the ventilated container than for the sealed one (36.81%; SD = 12.78 and 31.46%; SD = 19.76 respectively), though difference (ventilation vs. closed) was not significant (difference = 0.289 ± 0.512, z = 0.565, *p* = 0.572).

### 3.2. Experiment 2: To Evaluate the Effect of Density

**Figure 2:**
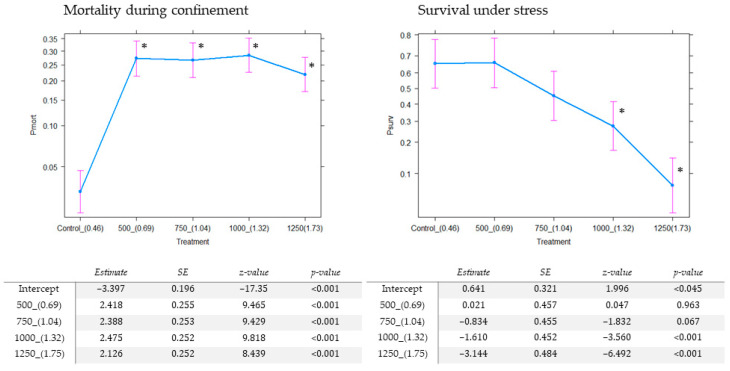
Effect estimates and standard errors derived from the adjusted model of different densities of mosquitoes on two parameters: Probability of death during confinement (Pmort, **left**) and probability of survival under stress (Psurv, **right**). Four densities of mosquitoes (number of pupae loaded and resting density of vertical surface in mosquito/cm^2^ between parentheses) evaluated against the control container. Table: parameter estimation for the effects of different densities of mosquitoes on two parameters with mixed model logistic regression. Asterisks indicate statistical significance compared with the control (α-level 0.05).

Average mortality during confinement was significantly higher in the new designs than in the control (Figure 2 and Table 1). The comparison of marginal means revealed that mortality across different densities did not differ with the new design, since the difference between the highest and lowest value was not significant (z = 1.56, *p* = 0.12).

Average survival under stress declined as density increased (Figure 2). The effects of the two lowest densities (DRSs of 0.69 and 1.04) were not significantly different from those of the control.

### 3.3. Experiment 3: Validation of the Chosen Design

Mortality during confinement in Experiment 3 was on average 3.8%, and it was 3.9% for the control container. No statistically significant differences were found between the new design and the control (Table 1).

**Figure 3:**
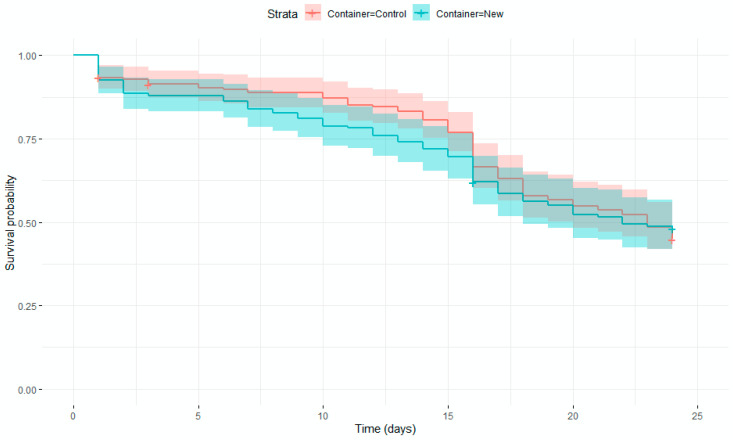
Kaplan–Meier plot to illustrate the survival of male mosquitoes in two different containers. Shaded areas show 95% confidence intervals. Crosses show censored individuals.

The Kaplan–Meyer plots for the different treatments are similar (Figure 3). The estimated average life was 18.7 days in the control (*n* = 197, SE = 0.5) and 17.6 days in the new design (*n* = 174, SE = 0.6). The hazard ratio for the new design was 0.995 as compared to the control, not significantly different from one (z = −0.03, *p* = 0.93). This suggests that no significant effect of container type was found.

## 4. Discussion

The first two experiments analyzed the most important design features of the release container. According to the results, lateral ventilation was deemed unnecessary as a design feature. There were no differences in mortality and resistance to stress after confinement in laterally ventilated and sealed containers. Vertical air flow was considered sufficient for this design, as long as the lower part remained on a ventilated surface, such as a grid tray. However, it must be highlighted that the need for ventilation was tested using a 12 cm high tube. It is possible that with longer tubes, vertical airflow may be insufficient.

The density of mosquitoes is one of the most important factors in the design of a release container. The greater quantity of mosquitoes able to be packed, the more efficient the release. However, overcrowding is known to have negative effects on the quality and longevity of mosquitoes [29,30]. For the design of the container, the release of small numbers of mosquitoes was considered, as several authors recommend low release rates at higher frequencies for the release strategy [31,32]. Low capacity containers allow easy dosing of release units. Optimal density will impact quality. For the purposes of this project, a similar quality to the ongoing pilot project was maintained. As a result, the quality levels of different densities in the AIO containers according to protocol were compared, since good results in the field have been reported (Tur et al. *in prep*.). According to the results, these AIO containers should be loaded with 500 pupae to guarantee a high male quality. However, large-scale projects would require the handling of large numbers of containers, so it is important to integrate their use into automated processes, such as automatic pupae dispensers and container conveyor belts.

The use of these containers at higher densities would require further field testing for future development.

The optimal DRS value is also similar to the optimum documented by Iyaloo et al. [33] for storage/release containers (0.55 male mosq./cm^2^). The effect of density on caged mosquitoes is believed to be related to the availability of vertical surfaces on which to rest. For this reason, density is usually measured in relation to the available vertical resting surfaces in the cage [26]. The proposed container maximizes the resting surface by the addition of eight vertical strips that account for 53% of the total estimated vertical resting surface (686 cm^2^). This increase in internal surface area permits a higher density of mosquitoes in a space one tenth of a standard manual release container, without compromising quality. The use of geometries that increase the internal surface area seems to be an effective means of reducing the volume of mosquito cages without losing quality. The findings by Zhang et al. [30] also support this, in which the performance of egg-producing cages was increased by the addition of internal panels.

The results of Experiment 2 in regard to mortality during confinement indicated that the new container design had a significant impact on mortality (23% increase), without taking density into consideration. These results could be interpreted as the measurable effect of the container or its handling protocol. In Experiment 3, the container was removed from the water 24 h earlier, with similar mortality results to both control tests in Experiments 2 and 3 (<4%). The fact that the lower part of the container was completely submerged may be interpreted as problematic for the males. In *Aedes* species, adults remain inactive for a few hours after emergence to stiffen the exoskeleton and wings [34]. Overcrowding could lead to an increase in mosquitoes falling into the water, causing them to become trapped by the surface tension of the water. Therefore, it would be advisable to minimize the time containers are kept partially submerged in water.

It can be concluded that the proposed container and protocol according to Experiment 3, is a suitable system in which to keep adult male mosquitoes to be released in rear-and-release control methods. The containers are loaded with a predefined number of male pupae after sex-sorting and/or sterilization. The males maturate and rest undisturbed inside the containers until final release in the field. They have access to water and sugar necessary to assure their survival and future field performance [21,35]. The design of the container is also compatible with an automated release system. Given that the inner structure is pushed in order to release the males, release could be operated by electronically-controlled pistons. Indeed, TRAGSA is currently developing an electronically-controlled terrestrial release system based on this container with similarities to the system used for aerial dosed release of *C. capitata* [36]. This system is able to release varying amounts of insects according to geographical position and features a configurable pattern of releases. It is therefore useful to carry out release strategies focusing on population density of the target pest [37] or other parameters as disease incidence. The proposed system is similar in concept to the release system proposed by Crawford et al. [17] in the release of 14.4 million *Wolbachia* infected *Ae. Aegypti* with remarkable success. This system succeeded in suppressing wild populations, supporting the idea that release automation with low adult handling is both efficient and feasible. The proposed method is based on a less sophisticated concept, using generally available and cost-efficient materials that allow for its adaptation to different contexts. In addition, the container can be used for the irradiation of pupae and adult in SIT programs and is suitable for small-scale SIT projects based on manual release, reducing the need of space without compromising male quality. A fully-automated release system could be installed in public transport routes (e.g., buses or refuse collection lorries) to reduce costs of release in SIT and related vector control programs.

## 5. Conclusions

In this article, we have demonstrated how mosquitoes can be maintained in relatively undisturbed conditions from pupal stage to release using a specifically designed container that can be used in an automated terrestrial release system for SIT and other related control methods. It can be concluded that this method saves time and space as compared to manual systems widely used in pilot projects whilst eliminating post-emergence handling associated with the majority of large-scale release systems.

## Figures and Tables

**Table 1:** Parameter estimation to compare mortality during confinement in a new container design and a control. Mortality during confinement.

	*Estimate*	*SE*	*z-Value*	*p-Value*
Intercept	−3.190	0.116	−27.432	<0.001
New design	−0.048	0.186	−0.255	<0.798

## Data Availability

Not applicable.
